# Reirradiation of Local Recurrences of Prostate Cancer: PROSTARE (PROstate Cancer STereotActic REirradiation) Early Safety Analysis of a Phase 2 Study with a Limited Cohort

**DOI:** 10.3390/cancers18050848

**Published:** 2026-03-06

**Authors:** Wojciech Majewski, Aleksandra Napieralska, Marcin Miszczyk, Anna Misiorowska-Gołosz, Marcela Krzempek, Małgorzata Stąpór-Fudzińska, Justyna Rembak-Szynkiewicz, Jerzy Wydmański

**Affiliations:** 1 Radiotherapy Department, Maria Sklodowska-Curie National Research Institute of Oncology, Gliwice Branch, 44-101 Gliwice, Poland; aleksandra.napieralska@gliwice.nio.gov.pl (A.N.); anna.misiorowska@gliwice.nio.gov.pl (A.M.-G.); 2 Department of Oncology, Faculty of Medical Sciences in Zabrze, Medical University of Silesia, 40-055 Katowice, Poland; 3 Radiotherapy Department, Maria Sklodowska-Curie National Research Institute of Oncology, Krakow Branch, 31-115 Krakow, Poland; 4 Department of Urology, Comprehensive Cancer Center, Medical University of Vienna, 1090 Vienna, Austria; marcin.miszczyk@meduniwien.ac.at; 5 Collegium Medicum—Faculty of Medicine, WSB University, 41-300 Dąbrowa Górnicza, Poland; 6 Department of Biostatistics and Bioinformatics, Maria Sklodowska-Curie National Research Institute of Oncology, Gliwice Branch, 44-101 Gliwice, Poland; marcela.krzempek@gliwice.nio.gov.pl; 7 Department of Radiotherapy and Brachytherapy Planning, Maria Sklodowska-Curie National Research Institute of Oncology, Gliwice Branch, 44-101 Gliwice, Poland; malgorzata.stapor-fudzinska@gliwice.nio.gov.pl; 8 Department of Radiodiagnostics, Maria Sklodowska-Curie National Research Institute of Oncology, Gliwice Branch, 44-101 Gliwice, Poland; justyna.rembak-szynkiewicz@gliwice.nio.gov.pl

**Keywords:** prostate cancer, local recurrence, reirradiation, stereotactic body radiotherapy, SBRT, salvage stereotactic body radiotherapy, sSBRT, salvage treatment, toxicity

## Abstract

**Objective**: This study aims to report the early safety outcomes from an ongoing single-center, non-randomized phase 2 trial on focal salvage stereotactic radiotherapy (s-SBRT) for local prostate cancer recurrence. **Materials and methods**: This prospective phase 2 study includes patients with local recurrence after conventional or hypofractionated radiotherapy, ultrahypofractionated radiotherapy, or post-prostatectomy radiotherapy. The present analysis includes an initial subset of 21 out of 55 planned patients. All patients undergo mpMRI and PSMA-PET; biopsy is not required if imaging results are unambiguous. Focal s-SBRT is delivered to the recurrent lesion with a dose of 5 × 6.75 Gy. The primary endpoint is the rate of treatment-related CTCAE v5.0 grade ≥ 3 genitourinary (GU) or gastrointestinal (GI) toxicity. Secondary endpoints include early biochemical response (BR), defined as any PSA decline at 3 months. **Results**: With a median follow-up of 14 months (range: 4.5–25), one patient (4.8%) experienced both early and persistent late Grade 3 GU toxicity (bladder bleeding). Late Grade 2 GU and GI toxicities occurred in five (23.8%) and one (4.8%) patients, respectively. In exploratory univariable analysis, PTV volume 13 cc was identified as a marginal predictor for increased GU/GI radiation reactions (*p* < 0.1). Regarding efficacy, all 21 patients (100%) demonstrated an early biochemical response, with 15 patients (71.4%) achieving a PSA reduction of 50%. **Conclusions**: Focal s-SBRT demonstrates a favorable early safety profile and consistent biochemical response, supporting the preliminary safety of this ongoing study.

## Simple Summary

The PROSTARE study is an ongoing prospective clinical trial investigating the safety of focal salvage stereotactic body radiotherapy (s-SBRT) for patients with local prostate cancer recurrence after previous radiation therapy. This preliminary analysis reports on the first 21 evaluable patients out of a planned total cohort of 55. The results so far show that the treatment is safe and effective; severe side effects (Grade 3) were rare, occurring in only one patient (4.8%), with no cases of severe complications like fistulas or necrosis. Interestingly, an exploratory analysis suggests that a larger treatment volume (PTV > 13 cc) might be a marginal predictor of increased side effects. Regarding efficacy, all patients (100%) experienced a decrease in PSA levels, with 71.4% achieving a reduction of more than 50%. These early results support the use of focal s-SBRT as a promising, low-toxicity treatment option, though longer follow-up is needed to confirm these findings.

## 1. Introduction

Approximately half of biochemical failures following definitive external beam radiotherapy (EBRT), and up to 20% after radical prostatectomy (RP) combined with prostate bed radiotherapy (RT), presents as local recurrence, with approximately 50% of these failures occurring as an isolated site [1,2,3,4,5]. The choice of treatment in those with local recurrence is challenging, and the risk of excessive toxicity should be taken seriously into account with any interventional treatment administered within a previously irradiated volume. Numerous retrospective studies and only few prospective ones have investigated the use of salvage stereotactic body radiation therapy (s-SBRT) for reirradiation in those patients. The results were also evaluated in pooled analyses, indicating a high level of tolerance and promising outcomes [6,7,8,9]. The MASTER meta-analysis authors determined that s-SBRT is more than likely safer than numerous alternative salvage treatments, including surgery, based on the available, primarily retrospective, evidence [10]. Nevertheless, interpretation was impeded by numerous significant limitations, including the short follow-up, the influence of publishing bias, and the extensive heterogeneity of endpoint definitions. Despite our initial positive experience with s-SBRT, it was later found to be associated with unacceptable toxicity. An analysis of a larger group of patients revealed that as many as 32% of the patients experienced grade ≥ 3 adverse events (AE) over a longer course of follow-up [11,12]. The irradiated volume was the primary risk factor for complications. Patients who underwent focal SBRT (i.e., SBRT for a recurrent lesion with a margin) had a 7.1% rate of severe or worse AE, compared to 44% for whole-gland reirradiation. Although this is not negligible, focal reirradiation is more reasonably balanced between risks and benefits in the context of recurrent disease. Furthermore, the ESTRO-ACROP consensus statement also recommends focal SBRT as a treatment approach, despite continued apprehensions regarding potential out-field intraprostatic progression [13]. Our subsequent investigation of s-SBRT in patients with local recurrence following post-operative radiotherapy, in whom whole-gland treatment was by definition impossible, further supported the hypothesis that reducing the volume of irradiation could result in a reduction in the incidence of severe adverse events [14]. In accordance with our expectations and clinical data, we discovered a G ≥ 3 toxicity rate of 6.3% following focal s-SBRT. This finding motivated us to conduct a prospective trial.

The majority of the studies on s-SBRT have been retrospective analyses conducted on small samples, with relatively few prospective ones as exceptions [15,16,17]. We refined our approach towards s-SBRT by utilizing the available evidence and our own experiences, and we established a phase 2 safety study called PROSTARE (PROstate cancer STereotActic REirradiation). It was our hypothesis that the reduction of the treatment volume with focal radiotherapy would result in a relatively low incidence of severe adverse events (AEs) while maintaining a reasonable level of oncologic efficacy. The preliminary safety data are detailed in this manuscript.

## 2. Materials and Methods

This study is being conducted at a single tertiary center, with the objective of evaluating the safety and efficacy of s-SBRT in prostate cancer patients. Eligible patients have a local recurrence following definitive EBRT (EBRT subgroup) or prostatectomy with post-operative irradiation (RP subgroup). The patients in the EBRT group are further divided based on the mode of fractionation used during the initial radiotherapy: conventional or moderate hypofractionation (EBRT subgroup A), or ultra-hypofractionation using fraction doses of ≥5 Gy (EBRT subgroup B). Focal salvage s-SBRT is administered to patients on a tumor that is visible in mpMRI/PET. The total dose is 33.75 Gy, with five fractions of 6.75 Gy delivered every other day. The fractionation schedule of 33.75 Gy in 5 fractions was adopted as the institutional standard for reirradiation. This represents a pragmatic dose reduction from the traditional 36.25 Gy (5 × 7.25 Gy) Stanford protocol, aimed at mitigating late toxicities observed in our earlier experience. This regimen provides a biologically effective dose (BED) of 185.6 Gy (assuming α/β = 1.5 Gy), which is comparable to other prospective salvage SBRT trials (e.g., 30–35 Gy in 5 fractions) while maintaining a safety margin for previously irradiated critical organs [18].

Eligible patients must have a local recurrence of prostate cancer following definitive or post-operative (adjuvant or salvage) radiotherapy that is confirmed by biopsy, or/and by consistent mpMRI and PET-PSMA findings and PSA growth dynamics. There must be a minimum of 2 years since primary radiotherapy, and good performance status (ECOG 0–1). Hormonal treatment either before or concurrently with s-SBRT is allowed. Patients are not eligible if they have polymetastatic disease (>5 lesions) or oligometastatic disease but are not suitable for local forms of metastasis-directed therapy (MDT). Tumor volume (GTV) > 14 cc, severe dysuria before s-SBRT (e.g., IPSS ≥ 19), and poor tolerability of primary radiotherapy (≥G3 toxicity) or persistent late toxicity ≥ G2 are also exclusion criteria. Patients with previous prostate brachytherapy are excluded as well.

### 2.1. Primary Endpoint

Our primary endpoint is treatment toxicity, defined as the rate of severe or worse (G ≥ 3) genitourinary (GU) and gastrointestinal (GI) radiation-induced toxicity early (3 months post-treatment) and late (2 years post-treatment), as measured by the CTCAE v5.0 criteria.

### 2.2. Secondary Endpoints

Secondary endpoints include rate of moderate or worse treatment toxicity, including early and late G ≥ 2 GU and GI radiation-induced toxicity. An early treatment outcome endpoint is biochemical response (BR), defined by the decrease in a PSA level below the baseline level before s-SBRT. There are also many other long-term endpoints: biochemical control (BC), metastasis-free survival (MFS), overall survival (OS), relapse-free survival (RFS), biochemical failure-free survival (BFS), local control (LC), and assessment of the patient’s stated quality of life based on the QLQ-C30 and PR-25 questionnaires.

### 2.3. Radiotherapy Simulation and Delivery

Diagnostic PSMA-PET and mpMRI of the prostate or prostate bed is performed and used for treatment planning. Fiducial implantation is not required. The patients are placed in a supine position and immobilized with thermoplastic masks. An empty rectum and partially empty bladder are recommended; however, the bladder may be filled to a volume of 200–300 mL if necessary, as was shown to be reproducible in our own study [19]. Treatment on a C-arm linear accelerator is preferred to CyberKnife. To verify the location of the gross tumor volume (GTV), CBCT must be conducted before each s-SBRT fraction.

The treatment target is restricted to focal reirradiation, which involves the irradiation of the visible tumor with an appropriate margin only. The GTV is defined as the tumor visible on mpMRI, and PET–CT will serve as an additional guidance of tumor location. The clinical target volume (CTV) is created by adding a 1–3 mm margin around the GTV. Finally, the planning target volume (PTV) is created by adding a uniform 3 mm margin to the CTV. In cases where the margin overlaps with the rectum and/or bladder, and high accuracy and reproducibility is ensured, it is possible to reduce PTV margins to 1 mm.

### 2.4. Dose Constraints

While the criteria for limiting the dose to nearby organs are not well-defined for reirradiation, the following doses should be aimed for: maximum rectal dose ≤ 103% of the prescribed dose (optimal ≤100%), D30% < 15 Gy, maximum bladder/urethra dose ≤ 105% of the prescribed dose (optimal ≤103%), D30% < 15 Gy.

### 2.5. Sample Size Calculation

A single-stage phase 2 single-arm clinical trial design (Fleming–A’Hern design) will be used to test whether the proportion responding (P) (i.e., without ≥G3 toxicity) warrants further investigation (H0: P ≤ p0 versus H1: P ≥ p1). For an α = 0.05, 1 − β = 0.9, the maximum response proportion of a poor treatment tolerance p0 = 0.75, and a good treatment minimum response rate p1 = 0.90, *n* = 55 subjects will be needed. The hypothesis that P ≤ 0.75 is rejected if the observed number of responses is 47 or greater, which would imply that treatment tolerance is satisfactory. The study will be terminated immediately if nine patients experience ≥ G3 GU or GI toxicity at any point during the investigation. This decision is due to safety and ethical reasons, because in such a situation the study objectives will not be met.

### 2.6. Statistical Analysis

Categorical variables will be presented as contingency tables, and continuous variables will be summarized using appropriate measures of central tendency and variability. The rate of ≥G3 GU and GI toxicity is reported, as well as the rate of ≥G2 toxicity. Univariable logistic regression analysis was performed regarding potential risk factors associated with genitourinary (GU) or gastrointestinal (GI) toxicity of any grade. These factors included age, total dose, volume of GTV and PTV, biopsy of recurrence, radiotherapy of regional lymph nodes, ADT use, and time to SBRT. For categorical variables, the category “no” was set as the reference. *p*-values were derived from likelihood ratio tests. Additionally, the early biochemical response is presented.

All intergroup comparisons will be performed using appropriate tests (parametric or non-parametric) with normality assessed via the Shapiro–Wilk test, and the relevant effect sizes will be calculated to quantify the magnitude of differences between groups.

The survival probability for secondary endpoints will be compared between subgroups using the log-rank test and visualized on Kaplan–Meier curves. S-SBRT efficacy will be examined in relation to treatment subgroups and selected prognostic factors, including cancer grade (ISUP grade), pretreatment PSA level, the use of hormonal treatment, the presence of concurrent oligometastatic disease, and castration resistance. To evaluate the impact of disease characteristics on event risk, the univariable and multivariable Cox proportional hazards model will be implemented. Hazard ratios (HRs) will be reported with 95% confidence intervals. Two-sided hypotheses will be tested, and statistical significance will be defined as a *p*-value of less than 0.05. The analyses will be conducted in R software v.4.5.1 (R Core Team (2025)) [20]. These secondary objectives will be assessed in a two-year and three-year period.

## 3. Results

From July 2023 to July 2025, 30 patients were enrolled in the study after giving their informed consent and received focal s-SBRT. A total of 21 patients underwent at least one follow-up visit beyond 3 months after sSBRT, making them evaluable for the early safety analysis.

Local recurrence was detected in 20/21 patients based on mpMRI and in 19/21 patients based on PSMA-PET imaging. A total of eight patients underwent a biopsy. Patients who did not have positive results on either imaging method had a biopsy-confirmed local recurrence. In 19 patients (90.5%), the prior radiotherapy plan has been registered in a planning system and fused with a planning CT.

Detailed patient characteristics are presented in Table 1. In addition, detailed information on treatment prior to reirradiation is provided in Table S1.

In three patients, the s-SBRT was the third irradiation administered within the prostate gland/prostate bed. These patients were included in the study because, according to Andrasche et al., the irradiated volumes between the second and third reirradiation did not overlap and were therefore classified as the second/third type of reirradiation [21].

In all patients, a C-arm accelerator was used with daily CBCT image guidance. While fiducial placements were not mandatory, the majority of patients (12/14) with intact prostates had fiducial/fiducials that remained from primary radiotherapy. The treatment was administered as per protocol to 19 patients; one patient had a minor (≤10%) deviation from the prescribed dose (31.25 Gy in 5 fx of 6.25 Gy), and one patient had a major deviation (30 Gy in 5 fx of 6 Gy). In both cases, the dose was decreased due to safety reasons (close adherence to the urethra/rectum).

**Figure 1:**
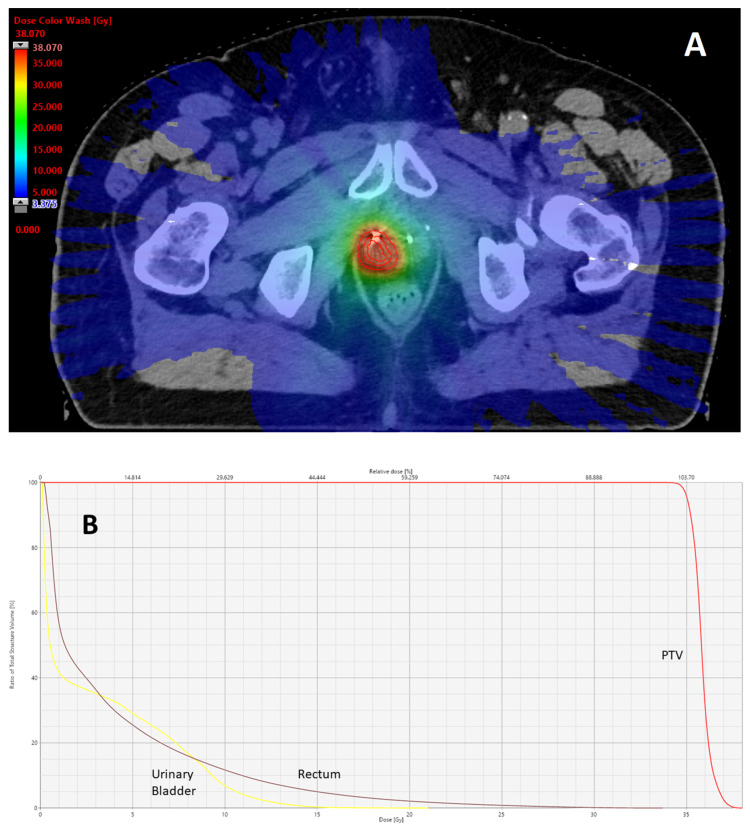
Dose distribution of focal s-SBRT (**A**) and dose volume histogram (**B**).

Figure 1 illustrates an example of the dose distribution of focal s-SBRT.

### 3.1. Early Safety Analysis

An early ≥G3 adverse event was observed only in one patient (bladder bleeding). Early G2 GU and GI adverse events were recorded in no patients and two patients, respectively.

All patients had a follow-up visit beyond 3 months post-sSBRT. With a median follow-up of 14 months (min–max: 4.5–25 months), only one patient had late ≥G3 toxicity. The persistent G3 GU toxicity was recorded in the same patient who experienced an early G ≥ 3 adverse event. Late G2 GU and GI adverse events were reported in five patients and one patient, respectively. No severe adverse effects, like fistula, necrosis, etc., were noted.

**Figure 2:**
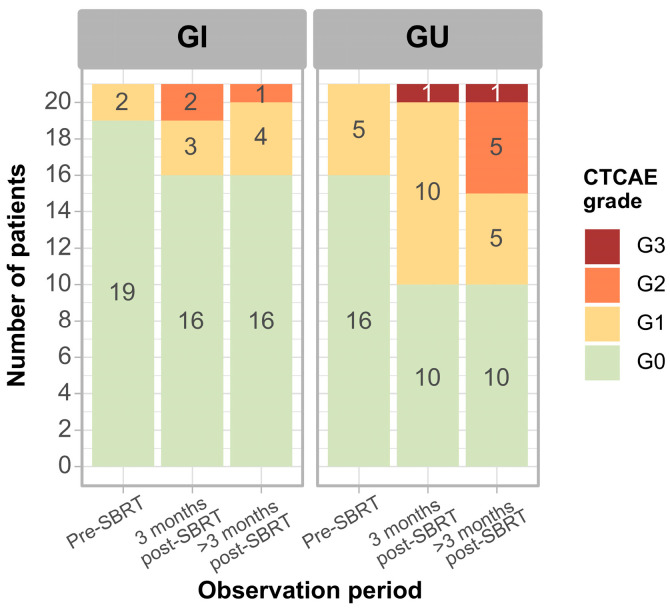
The cumulative incidence of CTCAE GU and GI maximum toxicity. Among the tested prognostic factors, only PTV volume above 13 cc marginally increased the risk of GI and GU radiation reactions (*p* < 0.1) (Table S2).

Figure 2 illustrates the maximum severity of adverse events by pre-treatment, early (until three months post-treatment), and late (between three months and the last follow-up), in accordance with CTCAE v5.0.

### 3.2. Biochemical Response

**Figure 3:**
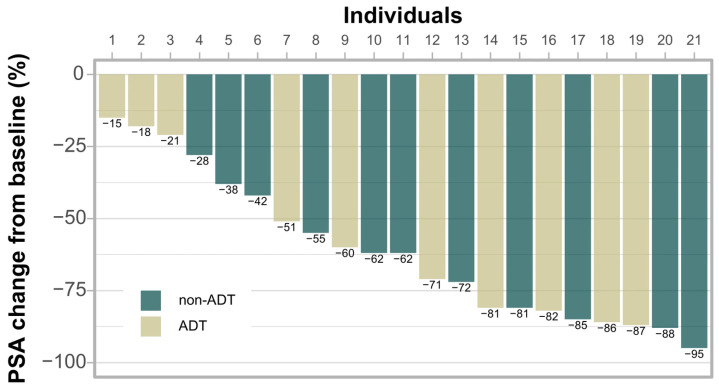
Early post-SBRT biochemical response (dots represent patients on ADT during s-SBRT).

Figure 3 shows the early BR at three months following s-SBRT. In all patients, a biochemical response, defined as any PSA decline, was observed, with 15 patients (71%) demonstrating a PSA reduction of >50%.

## 4. Discussion

The current study addressed a few critical issues. First, it utilized a focal SBRT aimed at improving treatment tolerance. There is an ongoing discussion of whether to use whole-gland or focal reirradiation [13,22]. Based on our earlier experience and ESTRO-ACROP recommendations, the choice of focal s-SBRT was well justified. The early safety analysis indicates that the treatment tolerance is good, with only one G3 adverse event. It is worth emphasizing that although this G3 adverse event was recorded, it may be considered as potentially reversible by medical intervention. For instance, Ekanger et al. [16] observed in their prospective study 16% late G3 GU toxicity, but the majority of these cases were resolved after medical interventions (including HBO treatment), with only 5% of severe durable toxicity. The rate of G2 toxicity in our study was also quite acceptable (24% GU, 5% GI AE). The toxicity rates reported by us align with established data from the literature [6,7,8,9,23]. In the largest retrospective database on reirradiation (RESTART), the rate of moderate and severe toxicity was 16% and 8% for GU and 5% and 4% for GI symptoms [22]. It is worth noting, however, that in prospective studies, the reported rate of adverse events should be more reliably scored. In the few prospective studies to date, the rate of G2 and G3 adverse events varies between 4–26% and 8–16% for GU and 8% and 4% for GI symptoms, respectively [15,16,17]. Our study has proceeded in accordance with its original design. From a clinical perspective, identifying factors that influence the risk of radiation toxicity is crucial for treatment optimization. For instance, Miszczyk et al. observed a clear association between irradiation volume and late toxicity [12]. Interestingly, our exploratory analysis revealed a similar trend toward statistical significance for PTV volume (<13 cc vs. >13 cc) regarding both GU and GI toxicity (*p* < 0.1). Although this did not reach the formal significance threshold, likely due to the limited sample size, it aligns with previous reports suggesting that larger treatment volumes may correlate with an increased risk of adverse effects. In the abovementioned prospective studies and in a multicenter observational longitudinal study (RESTART), various scales were used to assess radiation toxicity (CTCAE or RTOG), but the median follow-up was longer than in our study, ranging from 25 to 86 months.

Current therapeutic strategies for patients with local recurrence after radiotherapy remain a subject of clinical debate. The AUA/ASTRO/SUO guidelines outline several salvage local therapy options, including radical prostatectomy, cryoablation, HIFU, and reirradiation. Notably, they emphasize that the toxicity profile associated with various forms of reirradiation is significantly lower compared to other local salvage modalities [24]. Similarly, the EAU-EANM-ESTRO-ESUR-ISUP-SIOG guidelines acknowledge these options but maintain a more cautious stance regarding stereotactic body radiotherapy (SBRT) [25]. According to these recommendations, salvage SBRT should be offered primarily to selected patients in experienced centers, ideally as part of a clinical trial or a well-designed prospective study. Our findings align with these guidelines, suggesting that SBRT has a favorable safety profile.

There is a lack of reliable and widely accepted dose constraints for prostate reirradiation; therefore, our constraints may appear insufficiently robust. This reflects a broader limitation of contemporary studies in this field, as a clear consensus is still lacking (e.g., the ESTRO-ACROP consensus by Jereczek-Fossa et al., already cited in the manuscript).

We anticipate that further post hoc analyses after completion of the study—ideally incorporating detailed data from the first course of irradiation—may help to better define and refine dose constraints for prostate reirradiation.

The follow-up is still insufficient to reliably compare our findings with those from the literature, and the sample size of patients included in the study is too small to evaluate the tolerance of the predefined subgroups. The EBRT subgroup B (post-SBRT-failures) will be of particular importance, because SBRT is frequently applied as a standard primary treatment nowadays, and there is a lack of data in the literature on that specific subgroup. Recruitment for the study is proceeding better than anticipated, which is noteworthy given its status as a single-center study in a landscape of growing experience with s-SBRT in other centers across Poland.

The present study has several limitations that warrant consideration. First, we permit the inclusion of patients without biopsy confirmation, provided that their PSA is increasing and both MRI and PSMA-PET have indicated local recurrence, in contrast with recommendation statements [13,22]. We believe that this is appropriate from a clinical trial perspective, as PSMA-PET is a well-established and recommended method for detecting metastatic spread. In this scenario, there is no requirement for biopsy before MDT [26]. Although the possibility of false-positive results cannot be completely excluded, recent data on combined PSMA-PET and MRI demonstrate a high positive predictive value (up to 97.6%) for local recurrence, supporting the justification of our approach [27]. Nevertheless, we strongly advocate for a biopsy whenever feasible to ascertain the pathologic characteristics of a recurrent tumor. This procedure is mandatory for patients with inconsistent PSMA-PET and MRI results. It is important to mention that only 22% of ESTRO-ACROP consensus experts have considered a biopsy to be always necessary [13]. It is also worth emphasizing that all patients in our study responded to s-SBRT, even though a biopsy was performed on less than 50% of them. This indicates that the potential source of failure was accurately identified.

There are also other study limitations, which did not, however, influence this early safety analysis. Among these are the inclusion of CRPC, which is a more aggressive entity. However, local failure may respond to s-SBRT in a manner similar to metastases, such as delay in the initiation of systemic treatment or the development of new metastatic lesions or disease symptoms [28]. Hence, s-SBRT seems to have the potential to defer costly second-line systemic treatments and their associated side effects, or it might be administered as part of a multi-modality approach. Furthermore, the decision to include patients with oligometastatic disease may be questionable. According to the literature, more than half of the patients receiving MDT for oligometastatic prostate cancer remain free from further therapy after two years [29]. With a longer follow-up, this rate will probably drop substantially. If one assumes that MDT may be a curative treatment for that minority of patients [30], the concomitant MDT and local s-SBRT are quite reasonable. However, if we expect only prolongation of PFS, it may be questionable. We intend to compare the efficacy of s-SBRT in the group with oligometastases and in the group with solely local failures after the completion of the study. This comparison could determine whether any response is durable and whether s-SBRT should be considered in these patients. We recognize that the ADT permission is a confounding factor that will affect the interpretation of the results, at least as biochemically assessed endpoints. However, it should also be stressed that early biochemical response was achieved in all patients in our study irrespective of hormonal treatment, and it was even more pronounced in those without ADT (Figure 3). Nonetheless, the great majority of uro-oncology specialists believe that ADT is quite reasonable in patients with oligometastatic disease and would even implement it together with ARPI [31]. Furthermore, this analysis is limited by its relatively small sample size (*n* = 21) and the absence of a comparative control group. While the performed power analysis supports our primary findings, the small cohort may limit the ability to detect rare toxicities. In this context, our exploratory analysis suggested that PTV volume (>13 cc) was marginally significant (*p* < 0.1) for toxicity, but this requires validation in the full cohort of 55 patients. Additionally, the small median GTV (1.8 cc) indicates a highly select group of patients with early-detected recurrences, which may limit the generalizability of our findings to patients with more extensive local disease.

Finally, the current follow-up period remains relatively short. Given that radiation-induced complications, particularly those involving the bladder, often manifest several years after treatment, our findings primarily reflect acute and early-term tolerance. Continuous, long-term clinical monitoring of this cohort is ongoing to fully establish the late safety profile and to objectively validate the local control rates through long-term MRI-based radiological follow-up.

## 5. Conclusions

Herein, we report the initial safety and efficacy outcomes of the ongoing PROSTARE study. In this cohort of the first 21 evaluable patients, focal s-SBRT reirradiation for local recurrence was associated with a favorable safety profile; grade 3 adverse effects were limited to a single patient, with no cases of fistula or necrosis observed at a median follow-up of 14 months. These initial results support the hypothesis that limiting the reirradiation volume may significantly mitigate the risk of severe toxicity. Notably, our exploratory analysis suggests that PTV volume (13 cc) may be a marginal predictor of increased GU and GI radiation reactions, warranting careful treatment planning. Furthermore, the promising early biochemical response, with 71% of patients achieving a PSA reduction of 50%, suggests the potential efficacy of this approach. However, longer follow-up and the completion of the full study cohort are necessary to confirm these preliminary findings.

## Figures and Tables

**Table 1:** Patients’ characteristics.

Characteristic	Patients (*n* = 21)
Mean age (years)	69
Biopsy, *n*	
Yes	8
No	13
Type of recurrence, *n*	
Isolated locally	13
Local + oligometastatic	8
Concurrent ADT, *n*	
Yes	10
No	11
Hormonal status, *n*	
HSPC	16
CRPC	5
PSA doubling time (months), median (IQR)	10.6 (5.2–12.6)
Time from primary RT to local recurrence (months), median (IQR)	96.7 (59.2–151.1)
Pre-SBRT PSA (ng/mL), median (IQR)	1.32 (0.59–2.56)
GTV (cc), median (IQR)	1.8 (1.1–4.6)
Treatment subgroups, *n*	
EBRT A	9
EBRT B	5
RP	7

## Data Availability

The data presented in this study are available on request from the corresponding authors.

## Supplementary Materials

The following supporting information can be downloaded at: https://www.mdpi.com/article/10.3390/cancers18050848/s1, Table S1: Treatment of patients before s-SBRT; Table S2: Potential risk factors associated with adverse events.

## Abbreviations

The following abbreviations are used in this manuscript:

ADT	Androgen Deprivation Therapy
AE	Adverse Events
ARPI	Androgen Receptor Pathway Inhibitor
BC	Biochemical Control
BFS	Biochemical Failure-free Survival
CBCT	Cone-Beam Computed Tomography
CRPC	Castration-Resistant Prostate Cancer
CTCAE	Common Terminology Criteria for Adverse Events
CTV	Clinical Target Volume
ECOG	Eastern Cooperative Oncology Group performance status
EBRT	External Beam Radiation Therapy
G	Grade
HSPC	Hormone-Sensitive Prostate Cancer
LC	Local Control
MFS	Metastasis-Free Survival
mpMRI	Multiparametric Magnetic Resonance Imaging
MDT	Metastasis-Directed Therapies
OS	Overall Survival
PSMA-PET	Prostate-Specific Membrane Antigen Positron Emission Tomography
PTV	Planning Target Volume
RFS	Relapse-Free Survival
RP	Post-operative Radiotherapy
s-SBRT	Salvage Stereotactic Body Radiation Therapy
