# Supplementary material for: Reirradiation of Local Recurrences of Prostate Cancer: PROSTARE (PROstate Cancer STereotActic REirradiation) Early Safety Analysis of a Phase 2 Study with a Limited Cohort
Source: Cancers (Basel). 2026 Mar 6;18(5):848. doi: 10.3390/cancers18050848 (PMC12985271; doi:10.3390/cancers18050848)
Supplement: Supplementary file 1 [file cancers-18-00848-s001.zip › cancers-4153625-supplementary.pdf]

Table S1. Treatment of patients before s-SBRT.

Type of treatment	*Patients (N = 21)*
RP subgroup	7
Surgery	
Prostatectomy	5
Prostatectomy and lymph node dissection	2
ADT after surgery	
Yes	1
No	6
Time to first recurrence: med., range (months)	25.7 (1-80.2)
Salvage radiotherapy	
Conventional EBRT	6
SBRT	1
Time to second recurrence: med., range (months)	51.4 (26.7-148.6)
Subgroup A (Conventional EBRT)	9
ADT after radiotherapy	
Yes	4
No	5
Time to recurrence: med., range (months)	94.6 (21.4-176.1)
Subgroup B (SBRT)	5
ADT after radiotherapy	
Yes	1
No	4
Time to recurrence: med., range (months)	47.5 (35.4-88.1)

Table S2. Potential risk factors associated with adverse events.

	GI			GU		
Variable	*event/n (5/21)*	*OR [95%CI]*	*P*	*event/n (11/21)*	*OR [95%CI]*	*P*
Age	5/21	0.94 [0.77–1.12]	0.50	11/21	0.98 [0.84–1.14]	0.79
Total Dose	5/21	1.02 [0.96–1.1]	0.56	11/21	1.01 [0.96–1.06]	0.70
RTH of lymph nodes [Yes]	2/5	2.89 [0.29–27.37]	0.35	4/5	5.14 [0.59–113.42]	0.14
ADT use [Yes]	3/8	3.3 [0.42–31.89]	0.25	5/8	1.94 [0.33–13.04]	0.47
biopsy of recurrence [Yes]	2/8	1.11 [0.12–8.75]	0.92	4/8	0.86 [0.14–5.14]	0.86
GTV (continuous variable)	5/21	1.15 [0.83–1.6]	0.37	11/21	1.02 [0.76–1.41]	0.87
PTV (continuous variable)	5/21	1.07 [0.93–1.24]	0.31	11/21	1.03 [0.91–1.19]	0.65
PTV >13cc [Yes]	3/6	6.5 [0.77–71.14]	0.09	5/6	7.5 [0.91–164.04]	0.06
Time to SBRT	5/21	1.15 [0.89–1.56]	0.29	11/21	0.99 [0.79–1.24]	0.94
